# News and Notes

**Published:** 1907-07

**Authors:** 


### NEWS AND NOTES.

---

The College of Physicians and Surgeons of New York has just celebrated their one hundredth anniversary.

---

The commencement exercises of the University of N. C., were held on May 8th. Diplomas were given to 11.

---

The Baptist Memorial Sanitarium Association, Memphis, has applied for a charter to erect a building to cost $250,000.

---

Jefferson Medical College held its eighty-second annual commencement exercises June 3, when a class of 126 was graduated.

---

The annual meeting of the Association of Medical Officers of the Army and Navy of the Confederacy was held in Richmond, Va.

---

The governor of Pennsylvania has approved the bill appropriating $600,000 for the establishment and maintenance of a sanatorium for poor consumptives.

---

Government returns show that the deaths from the plague throughout India, for the six weeks ended May 11, reached 451,892. In the Punjab alone 286,777 deaths occurred.

---

The North Carolina State tuberculosis commission has purchased a farm of 800 acres in Moore County, on which the State Tuberculosis Hospital, authorized at the recent session of the legislature, will be located.

---

An organization known as the Emory Club was organized March 21 by the students of the Atlanta School of Medicine. The club is composed of graduates of Emory College and will work in the interest of that college and of the Atlanta School of Medicine.

---

The City Board of Health of New Orleans by a recent resolution has placed tuberculosis on the list of reportable diseases. The matter has excited considerable criticism and discussion in the daily press, but the weight of opinion seems to favor the action of the City Board.

---

The legislature of Nebraska is considering the adoption of a bill to compel Christian Scientists to pass state board examinations in physiology, hygiene, bacteriology, pathology, symptomatology, and diagnosis, and to limit their practice to diseases without the province of the surgeon.

---

A New York coroner's jury in the inquest on the death of Mrs. Corinne McBride, who died recently under the treatment of Eddyites, returned a verdict that the victim had died of acute lobar pneumonia, from which she might have recovered had she had proper medical attendance.

---

The cable states that the police in Berlin are making war on moving picture shows as medical authorities assert that they are injurious to the eyes. There are 200 cinematograph theaters in Berlin and its suburbs. The incessant movement of the films is regarded as especially harmful for children's eyes.

---

The board of trustees of the Grady hospital has passed a resolution introduced some time ago, giving to the present members of the visiting medical staff the privilege of naming associates, these appointments subject to the approval of the board. By this action the board of trustees virtually doubles the medical staff.

---

Once a year in Copenhagen several thousand mite boxes are distributed to responsible young women and they patrol the streets soliciting contributions from every passerby. Nearly $30,000 has been collected in this way for the children's hospitals and asylums during the four years since the custom was inaugurated.

---

Mr. Whitely, the London merchant who recently died under tragic circumstances, has bequeathed a large portion of his estate for charitable purposes. Nine London hospitals have benefited by substantial legacies, and the sum of $5,000,000 has been left to found, provide and maintain homes for the aged poor.

---

The following officers have been elected by the Alabama Medical Society: President, Dr. S. W. Welch, of Talladega; senior vice-president, Dr. A. J. Caley, of Alexander City; junior vice-president, Dr. Henderson, of Troy; secretary, Dr. J. Norment Baker, of Montgomery; treasurer, Dr. H. G. Perry, of Greensboro.

---

Vaccination has again been upheld by a recent Massachusetts decision in a case brought by plaintiffs to test the legality of excluding a minor from the public schools because of failure to comply with the school committee's regulations. After passing through several courts the Supreme Judicial Court of the State decided for the defendant, that the minor must be vaccinated before being permitted to attend school.

---

The following officers were elected at the recent convention of the American Medical Association at Atlantic City: President, Dr. H. L. Burrell, of Boston; first vice-president, Dr. Edwin Walker, of Evansville, Ind.; second vice-president, Dr. Hiram R. Burton, of Lewes, Del.; third vice-president, Dr. G. W. Crile, of Cleveland, Ohio; fourth vice-president, Dr. W. B. Stewart, of Atlantic City; secretary, Dr. G. H. Simmons, of Chicago; treasurer, Dr. Frank Billings, of Chicago; members of the board of trustees, Dr. J. T. Happel, of Tennessee, Dr. W. W. Grant, of Colorado, and Dr. Philip Marvel, of New Jersey, all re-elected. The next meeting will be held at Chicago.

